# The Relevance of Reperfusion Stroke Therapy for miR-9-3p and miR-9-5p Expression in Acute Stroke—A Preliminary Study

**DOI:** 10.3390/ijms25052766

**Published:** 2024-02-27

**Authors:** Daria Gendosz de Carrillo, Olga Kocikowska, Małgorzata Rak, Aleksandra Krzan, Sebastian Student, Halina Jędrzejowska-Szypułka, Katarzyna Pawletko, Anetta Lasek-Bal

**Affiliations:** 1 Department of Physiology, Faculty of Medicine, Medical University of Silesia in Katowice, 40-752 Katowice, Poland; olga.kocikowska@sum.edu.pl (O.K.); hszypulka@sum.edu.pl (H.J.-S.); kpawletko@sum.edu.pl (K.P.); 2 Department of Histology and Cell Pathology, Faculty of Medical Sciences in Zabrze, Medical University of Silesia in Katowice, 40-752 Katowice, Poland; 3 Department of Engineering and Systems Biology, Faculty of Automatic Control, Electronics and Computer Science, Silesian University of Technology, 44-100 Gliwice, Poland; sebastian.student@polsl.pl; 4 Department of Neurology, School of Health Sciences, Medical University of Silesia in Katowice, 40-752 Katowice, Poland; aleksandra.krzan@sum.edu.pl (A.K.); alasek@gcm.pl (A.L.-B.); 5 Department of Neurology, Upper-Silesian Medical Center of the Silesian Medical University, 40-752 Katowice, Poland; 6 Biotechnology Centre, Silesian University of Technology, 44-100 Gliwice, Poland; 7 Department for Experimental Medicine, Medical University of Silesia in Katowice, 40-752 Katowice, Poland

**Keywords:** stroke, reperfusion treatment, miRNA, NGS, hsa-miR-9-3p, hsa-miR-9-5p, enrichment analysis, GO, KEGG

## Abstract

Reperfusion stroke therapy is a modern treatment that involves thrombolysis and the mechanical removal of thrombus from the extracranial and/or cerebral arteries, thereby increasing penumbra reperfusion. After reperfusion therapy, 46% of patients are able to live independently 3 months after stroke onset. MicroRNAs (miRNAs) are essential regulators in the development of cerebral ischemia/reperfusion injury and the efficacy of the applied treatment. The first aim of this study was to examine the change in serum miRNA levels via next-generation sequencing (NGS) 10 days after the onset of acute stroke and reperfusion treatment. Next, the predictive values of the bioinformatics analysis of miRNA gene targets for the assessment of brain ischemic response to reperfusion treatment were explored. Human serum samples were collected from patients on days 1 and 10 after stroke onset and reperfusion treatment. The samples were subjected to NGS and then validated using qRT-PCR. Differentially expressed miRNAs (DEmiRNAs) were used for enrichment analysis. Hsa-miR-9-3p and hsa-miR-9-5p expression were downregulated on day 10 compared to reperfusion treatment on day 1 after stroke. The functional analysis of miRNA target genes revealed a strong association between the identified miRNA and stroke-related biological processes related to neuroregeneration signaling pathways. Hsa-miR-9-3p and hsa-miR-9-5p are potential candidates for the further exploration of reperfusion treatment efficacy in stroke patients.

## 1. Introduction

Stroke is one of the leading causes of death and long-term disabilities in adults worldwide. The Global Burden of Diseases report states that the number of strokes increased worldwide between 1990 and 2019, particularly in the population of patients under the age of 65 [1]. The modern reperfusion treatment (RT) of ischemic stroke allows for lysis and/or the removal of thrombus from the closed lumen of the carotid or cerebral arteries via intravenous thrombolysis with the *IV* RT-plasminogen activator (rt-PA), and/or mechanical thrombectomy (MT). These methods increase the likelihood of penumbra reperfusion, which occurs in the ischemic region around the heart of the brain infarct. RT (i.e., IV rt-PA and MT) aims to preserve as many hypoxic cells as possible in this zone, including neuronal, endothelial, and glial cells.

In 2015, a randomized trial confirmed the efficacy and safety of MT in patients with stroke. Notably, 3 months after performing MT in the acute phase of stroke, 46% of patients were living independently, compared to 26.5% in the conservative treatment group from the trial [2]. Recent studies and meta-analyses have highlighted MT as an important treatment modality for stroke due to its potential to increase the length of the therapeutic window [3,4]. Now, physicians perform MT up to 24 h after stroke onset, according to the patient’s clinical and radiological profile, improving their chances of clinical success.

Based on the results of the meta-analysis, the use of MT in stroke patients results in the recanalization of more than 80% of the arteries that underwent the intervention and a return to full patient independence in approximately 45% of patients within 3 months after stroke. However, this means that a significant post-stroke neurological deficit remains in half of the patients, including some of those who have favorable angiographic results after MT. This reveals that a significant proportion of patients have futile recanalization (as their angiogram showed recanalization). There are likely many factors that influence the clinical outcome of thrombectomy. The unfavorable prognostic parameters identified in the subpopulation of endovascularly treated stroke patients include the following: older age, diabetes mellitus, severe neurological deficit on the first day of stroke, and a low ASPECTS scale score [5,6,7,8]. Regardless, up to 9% of patients reocclude within 48 h after complete recanalization. Identifying parameters that reduce the clinical benefit of MT can improve therapeutic strategies and ultimately better guide stroke reperfusion treatment. The search for the optimal qualifying tools for stroke treatment has been ongoing for years. Using patients’ miRNA profiles when selecting them for MT might help neurologists reduce the risk of failure and improve the clinical effects of MT.

In the age of emerging personalized medicine, biochemical and molecular biomarkers are being investigated to improve prognostic value with respect to the course of stroke and post-stroke disability. Therefore, many studies are focused on discovering miRNAs for use as potential biomarkers for stroke diagnosis and prognosis. Most of them involve the extraction of differentially expressed miRNAs (DEmiRNAs) from large-scale expression profiles, such as next-generation sequencing (NGS) data. The discovery of DEmiRNAs allows miRNAs with significant changes in expression levels, which have the potential to become biomarkers, and those with insignificant changes to be distinguished. Therefore, by examining the patient’s miRNA profile in the first hours and days after ischemic stroke, one could predict which processes (i.e., neuroprotection or neurodegeneration) will predominate in that patient [9,10,11,12,13]. Understanding specific molecular processes associated with restoring blood flow in hypoxic areas and the mechanisms underlying reperfusion injury could help predict clinical deficits in patients who have suffered an ischemic stroke.

The presented study is the first to report on changes in miRNA expression in the acute phase of stroke in patients who have undergone endovascular treatment. The clinical status of patients can change over several hours and days after the onset of a stroke. Therefore, we analyzed how reperfusion treatment affects the miRNA profile 10 days after stroke onset. Next, we used the results of the statistical NGS data to search for target genes of DEmiRNAs, which we validated using qRT-PCR. Then, we performed a functional analysis of the target genes to find out which metabolic pathways they are involved in. To identify and understand the biological role of miRNAs, we analyzed how reperfusion-induced changes in the miRNA expression profile might affect estimated targets (ETs) and how these changes might relate to ischemic stroke treatment.

## 2. Results

### 2.1. Study Design

**Figure 1:**
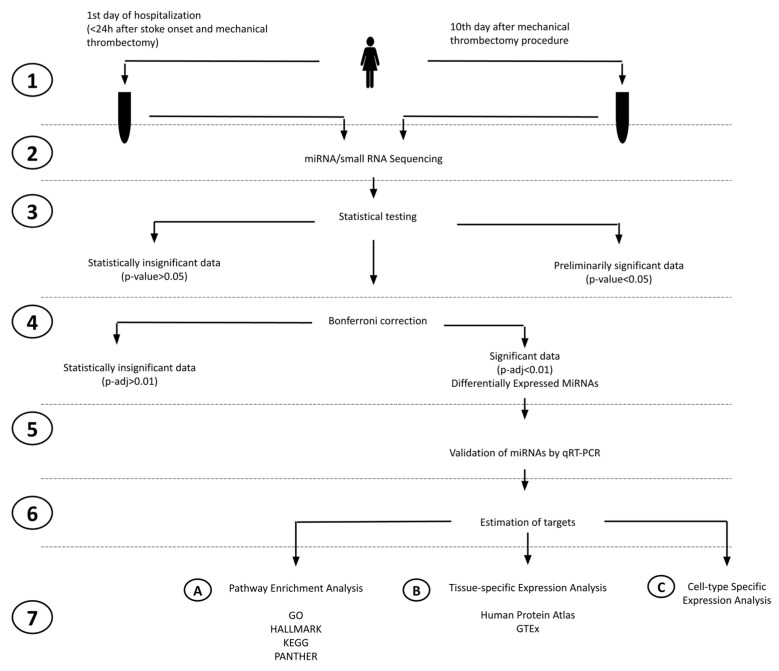
Study overview flowchart. The flowchart illustrates the key steps of our study as follows: (1) blood collection and serum separation, (2) miRNA/small RNA sequencing, (3) statistical testing (Exact Test), (4) Bonferroni correction, (5) miRNAs using qRT-PCR on a larger patient group and the statistical testing of qRT-PCR data, (6) estimation of gene targets based on MSigDB, (7A) GSEA (gene set enrichment analysis), (7B) TSEA (Tissue-Specific Expression Analysis), and (7C) CSEA (cell-type-specific expression analysis).

We analyzed circulating miRNA in serum samples from stroke patients on days 1 and 10 after stroke onset to find differentially regulated miRNAs and perform the enrichment analysis of significant miRNA gene targets. Figure 1 presents the study design. Table 1 summarizes the detailed clinical characteristics of the patients included in the NGS study, and Table 2 summarizes the detailed clinical characteristics of the patients included in the RT-qPCR validation study.

### 2.2. NGS miRNA Discovery

**Figure 2:**
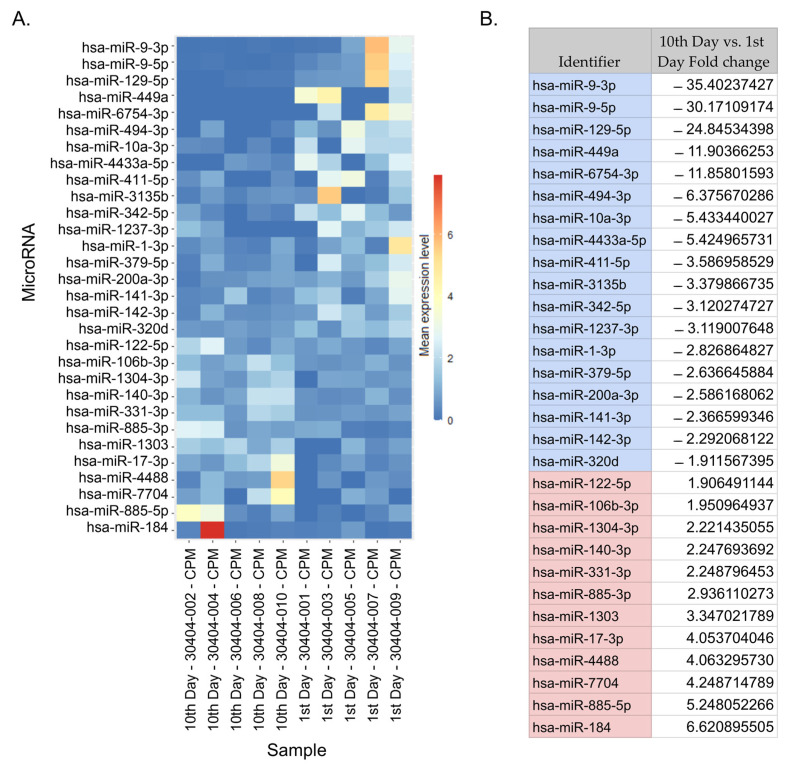
DEmiRNAs. (**A**) A heatmap depicting preliminary significant miRNAs based on a normalized expression. The heatmap shows the fold change in miRNA expression from the lowest to highest for samples between day 10 after reperfusion and day 1 after stroke onset. The sample description indicates the time of sample collection (i.e., an odd number indicates the sample was collected on day 1 post-stroke, and an even-numbered sample was collected on day 10). (**B**) A table listing the preliminary significant miRNAs and their fold change values. The top three scores in the table are the following DEmiRNAs: hsa-miR-9-3p, hsa-miR-9-5p, and hsa-miR-129-5p.

**Figure 3:**
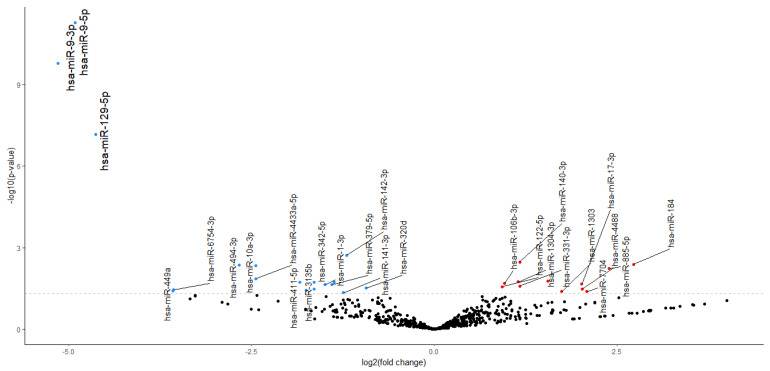
Volcano plot. Expression changes in preliminary significant miRNAs are presented in the volcano plot. Red dots indicate increased expression (12 preliminary significant miRNAs), and blue dots indicate decreased expression (18 preliminary significant miRNAs). Larger font indicates the following 3 significant DEmiRNAs: hsa-miR-9-5p, hsa-miR-3-5p, and hsa-miR-129-5p.

We used NGS analysis to find DEmiRNAs in the serum of stroke patients between day 10 after the reperfusion procedure and day 1 after stroke onset. Because of the small sample set mentioned above and the variations that might be due to individual differences, we assumed errors in categories I and II (the occurrence of false positives and false negatives). Therefore, we applied a larger confidence range to avoid potentially removing significant preliminary data. We found 30 preliminary significant miRNAs (*p* < 0.05) in a dataset of 2632 miRNAs. In total, 18 miRNAs had an upregulated expression, and 12 had a downregulated expression (Figure 2). After correction for multiple tests (Bonferroni correction, *p*-adjusted < 0.05) 3 significant and downregulated miRNAs emerged from 30 preliminary significant miRNA: hsa-miR-9-3p (*p* = 1.78 × 10^−7^), hsa-miR-9-5p (*p* = 5.54 × 10^−9^), and hsa-miR-129-5p (*p* = 7.37 × 10^−5^). These miRNAs were used in further target prediction and enrichment analysis (Figure 3).

### 2.3. DE-miRNAs Validation

**Figure 4:**
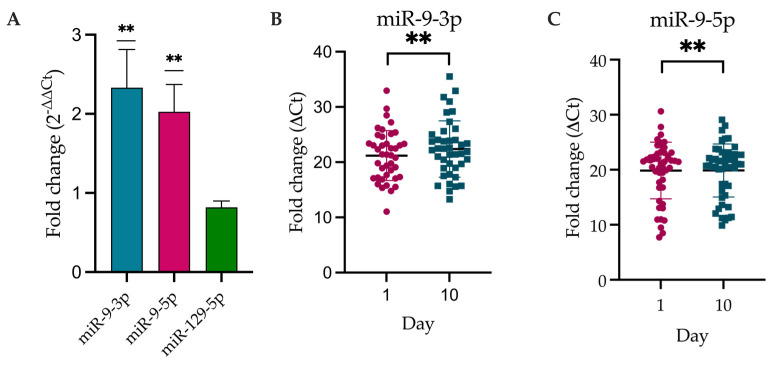
Expression level of DEmiRNAs according to Livak’s method. (**A**) Histogram representing the fold change, 2^−ΔΔCt^, for validated miRNAs. (**B**,**C**) Dot plots representing individual values for miR-9-3p and miR-9-5p, the level of which was significantly different between day 10 (turquoise dots) and day 1 (magenta dots) (normalized against UniSp6). ΔCt = Ct (target miRNA) − Ct (control miRNA); ** *p*-value < 0.01 (one sample *t*-test, µ = 1), miR-9-3p *p*-value = 0.0087 and miR-9-5p *p*-value = 0.0046.

We performed the validation of the following three significantly downregulated DEmiRNAs from NGS statistical analysis: miR-9-3p, miR-9-5p, and miR-129-5p (Figure 4). The levels of miR-9-3p and miR-9-5p significantly decreased between day 10 and day 1 after stroke. However, the miR-129-5p level did not reach statistical significance.

**Figure 5:**
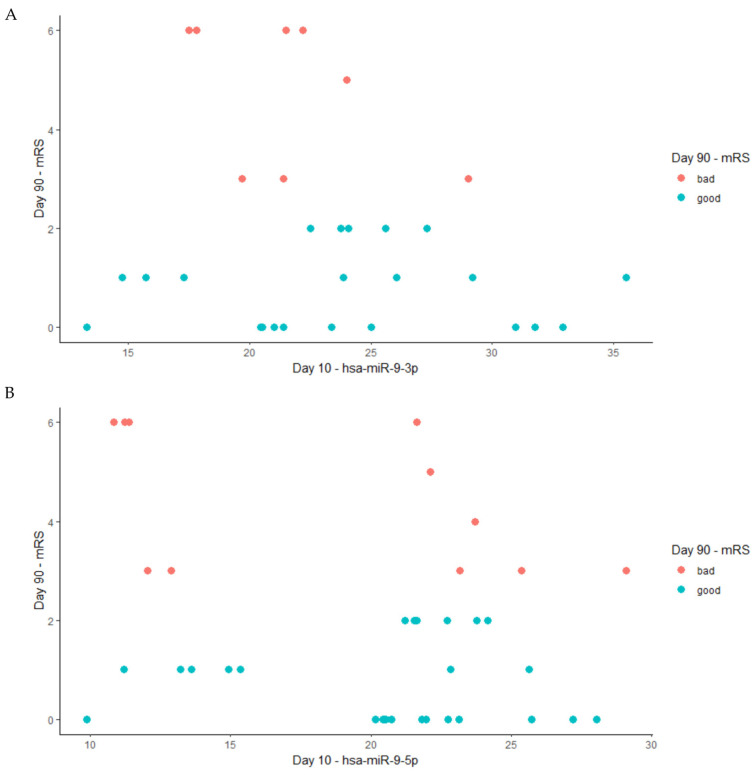
The Spearman correlation between the levels of miRNA on the 10th day after a stroke and the patient’s functional outcome on day 90, where red dots present data for patients with bad functional outcomes based on the mRS (3–6) and blue dots present data for patients with good functional outcomes based on mRS (0–2). (**A**) hsa-miRNA-9-3p (Spearman, r = −0.13); (**B**) hsa-miR-9-5p (Spearman, r = −0.15).

The results of the Spearman correlation are presented in Figure 5. We did not find any significant correlation between the level of miR-9-3p (r = −0.13) or miR-9-5p (r = −0.15) on day 10 after stroke occurrence and the patient’s functional status on day 90 following a stroke as per the mRS scale [14].

### 2.4. miRNA Target Prediction for DEmiRNAs

**Figure 6:**
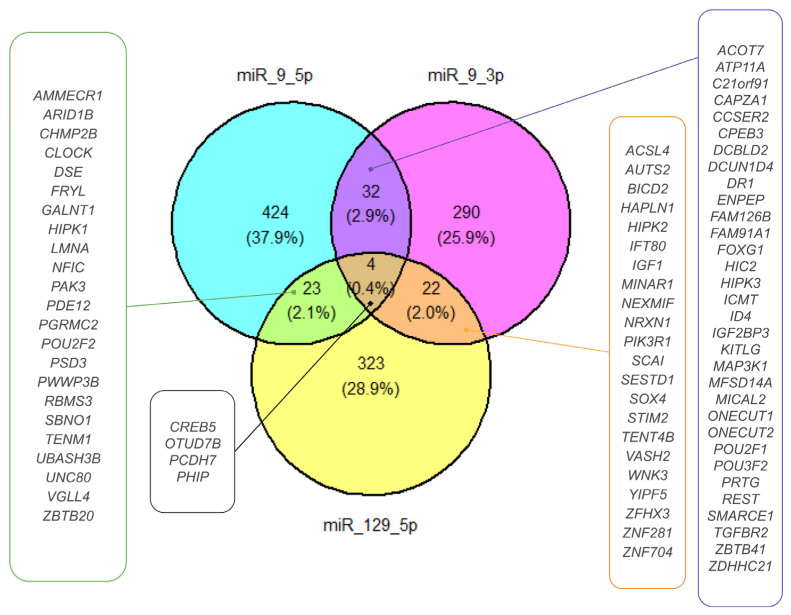
DEmiRNA ETs. The Venn diagram presents the ETs selected for DEmiRNAs. Targets shared by miR-9-5p and miR-129-5p, miR-9-3p and miR-129-5p, and miR-9-3p and miR-9-5p are shown in green-, orange-, and purple-framed boxes, respectively. Targets shared by miR-9-3p, miR-9-5p, and miR-129-5p are presented in a black-framed box.

Based on the target prediction analysis in the MSigDB database, we distinguished 1117 gene targets affected by DEmiRNAs [15]. Then, we restricted the number of targets to those shared between DEmiRNAs (Figure 6). For hsa-miR-9-5p and hsa-miR-9-3p, we found the following 32 common targets: ACOT7, ATP11A, C21orf91, CAPZA1, CCSER2, CPEB3, DCBLD2, DCUN1D4, DR1, ENPEP, FAM126B, FAM91A1, FOXG1, HIC2, HIPK3, ICMT, ID4, IGF2BP3, KITLG, MAP3K1, MFSD14A, MICAL2, ONECUT1, ONECUT2, POU2F1, POU3F2, PRTG, REST, SMARCE1, TGFBR2, ZBTB41, ZDHHC21. The hsa-miR-129-5p and hsa-miR-9-3p shared the following 22 common targets: ACSL4, AUTS2, BICD2, HAPLN1, HIPK2, IFT80, IGF1, MINAR1, NEXMIF, NRXN1, PIK3R1, SCAI, SESTD1, SOX4, STIM2, TENT4B, VASH2, WNK3, YIPF5, ZFHX3, ZNF281, ZNF704. Hsa-miR-129-5p and hsa-miR-9-5p shared 23 common targets: AMMECR1, ARID1B, CHMP2B, CLOCK, DSE, FRYL, GALNT1, HIPK1, LMNA, NFIC, PAK3, PDE12, PGRMC2, POU2F2, PSD3, PWWP3B, RBMS3, SBNO1, TENM1, UBASH3B, UNC80, VGLL4, ZBTB20). Hsa-miR-9-3p, hsa-miR-9-5p, and hsa-miR-129-5p shared the following four common targets: CREB5, OTUD7B, PCDH7, and PHIP. For further DEmiRNA gene target analysis, we chose four common targets, CREB5, OTUD7B, PCDH7, and PHIP, because gene co-targeting analyses show that miRNAs synergistically regulate cohorts of genes that participate in similar processes [15,16].

Given the large absolute values of DEmiRNA fold changes and their impact on previously mentioned targets, we used all targets in the enrichment analysis.

### 2.5. Pathway Enrichment for DEmiRNAs’ Targets Based on GO, HALLMARK, KEGG, and PANTHER Databases

**Figure 7:**
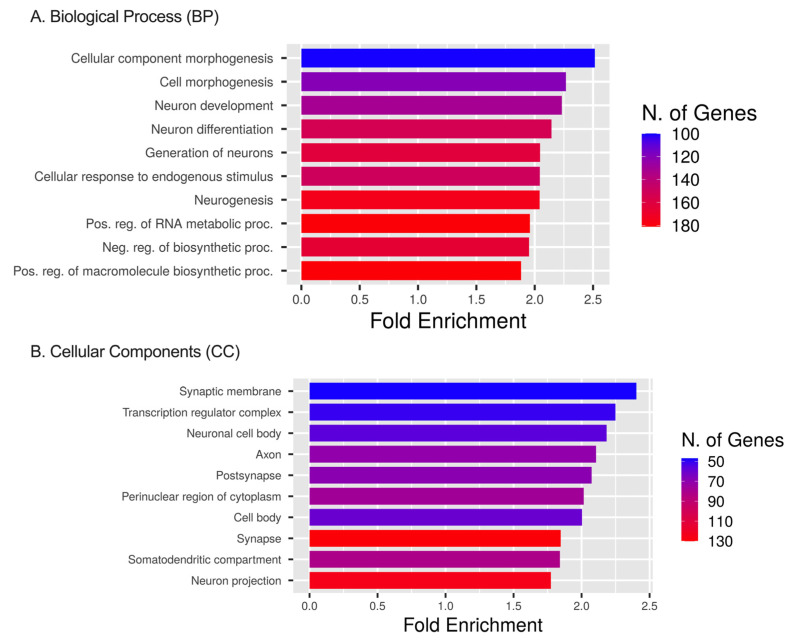
GO, BP, and CC analyses. (**A**) GO enrichment analysis of target genes regulated by significant DEmiRNAs. The diagram presents the top 10 target gene terms in BP pathways. (**B**) GO enrichment analysis of target genes regulated by significant miRNAs. The diagram presents the top 10 target gene terms in CC signaling pathways [16].

Analysis using the GO database showed that the ETs and DEmiRNAs were enriched in biological processes (BP), including cellular component (CC) morphogenesis, cell morphogenesis, neuron development, neuron differentiation, the generation of neurons, cellular responses to endogenous stimulus, and neurogenesis pathways (Figure 7A). Enrichment results identified up to 180 genes affected in these signaling pathways due to the DEmiRNAs. For CCs, the ETs were enriched in the transcription regulator complex, the perinuclear region of cytoplasm, and cell body pathways with up to 130 genes affected by DEmiRNAs (Figure 7B). We observed the effect of DEmiRNAs on their targets in the synaptic membrane, the neuronal cell body, the axon, and the post-synapse cellular compartment.

**Figure 8:**
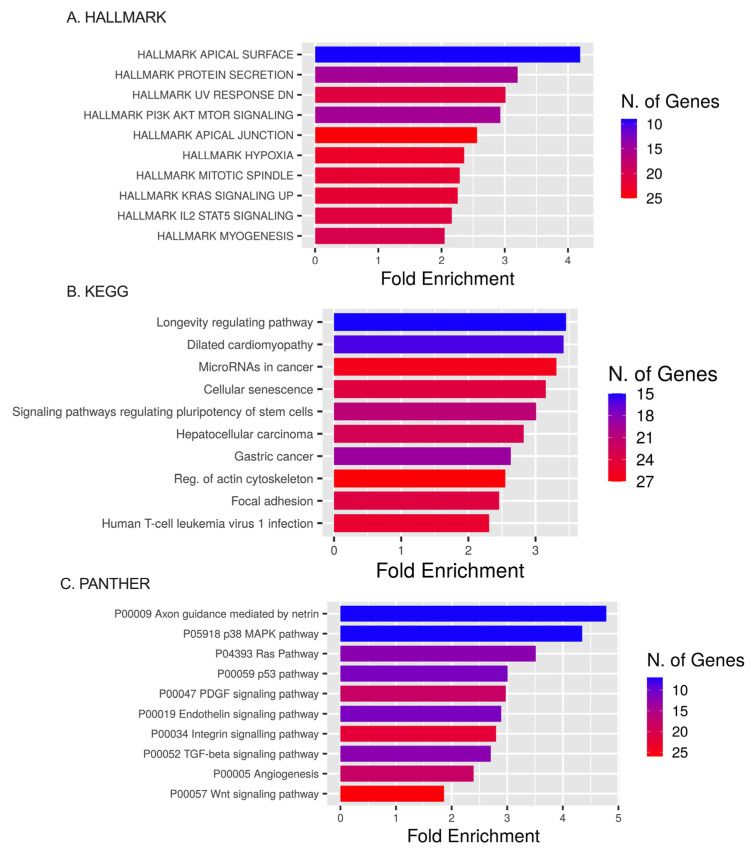
HALLMARK, KEGG, and PANTHER analyses. (**A**) The top 10 target gene terms in BP pathways for the HALLMARK enrichment analysis of target genes regulated by DEmiRNAs. (**B**) The top 10 target gene terms in CC pathways for the KEGG enrichment analysis of target genes regulated by significant miRNAs. (**C**) The top 10 target gene terms for the PANTHER enrichment analysis of target genes regulated by significant miRNAs.

Pathway enrichment analysis using the HALLMARK database showed that ETs primarily augmented in HALLMARK were the apical surface, protein secretion, UV response DN (down-regulated genes), apical junction, PI3K AKT MTOR signaling, hypoxia, and mitotic spindle pathways. DEmiRNAs’ targets were present within KRAS signaling up (genes up-regulated by KRAS activation), IL2 STAT5 signaling, and the myogenesis pathways (Figure 8A).

Pathway enrichment analyses using the KEGG database showed that ETs were expressed in the longevity-regulating pathway, dilated cardiomyopathy, miRNAs in cancer, cellular senescence, and signaling pathways regulating the pluripotency of stem cells (Figure 8B).

Pathway enrichment analysis using the PANTHER database showed DEmiRNA targets involved in axon guidance mediated by netrin, the p38 MAPK pathway, Ras pathway, p53 pathway, PDGF signaling pathway, endothelin signaling pathway, integrin signaling pathway, TGF-beta signaling pathway, and angiogenesis pathway (Figure 8C).

### 2.6. Tissue-Specific Expression Analysis (TSEA) and Cell-Type-Specific Expression Analysis (CSEA)

**Figure 9:**
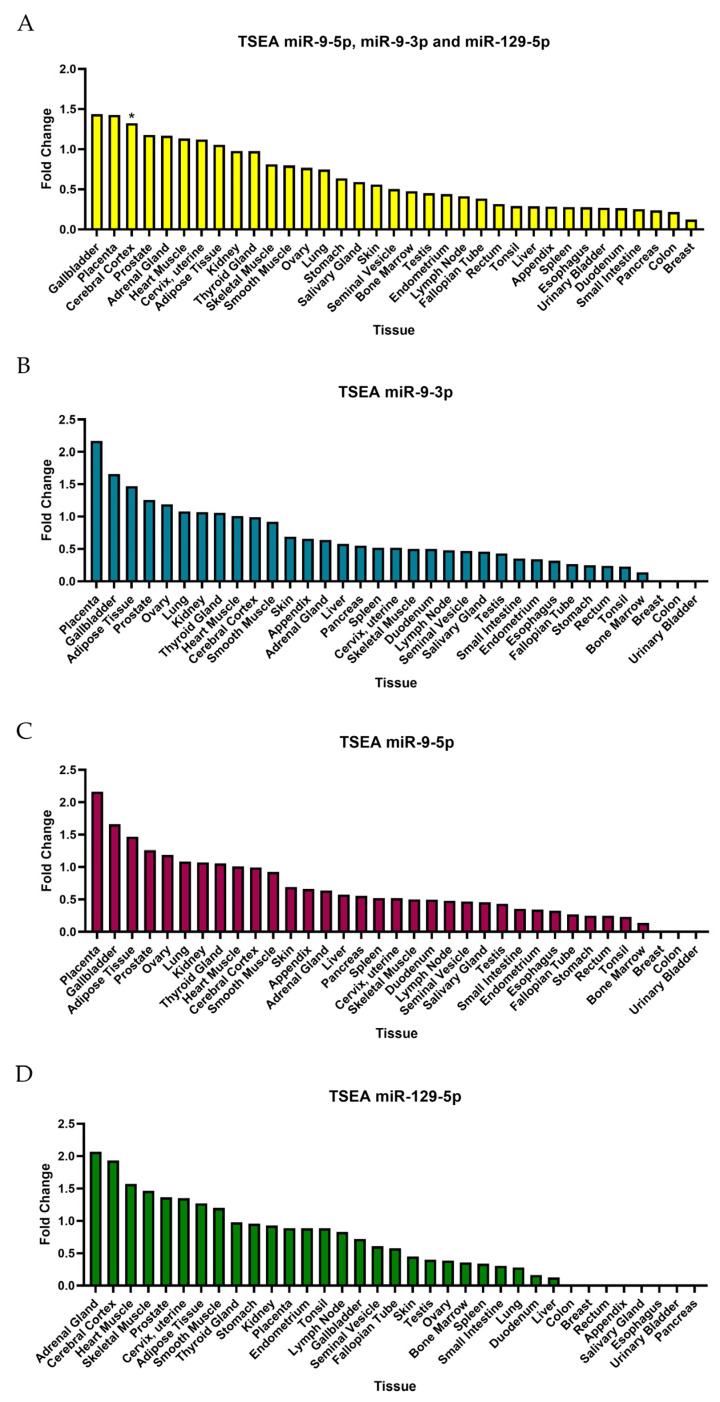
TSEA based on Human Protein Atlas. (**A**) Tissue-specific expression for all three DEmiRNAs. (**B**) TSEA for miR-9-3p. (**C**) TSEA for miR-9-5p. (**D**) TSEA for miR-129-5p. * The data show significance (*p*-value < 0.05) for summed targets of DEmiRNAs for the cerebral cortex; however, no data showed the significance of TSEA for separate targets of miRNAs.

TSEA for DEmiRNA targets based on the Human Protein Atlas for pooled targets of miR-9-3p, miR-9-5p, and miR-129-5p (Figure 9A) demonstrated a fold change value greater than one in the gallbladder, placenta, cerebral cortex, prostate, adrenal gland, heart muscle, cervix, uterine and adipose tissue, which referred to weak enrichment. However, the Benjamin–Hochberg correction only demonstrated significant enrichment in the cerebral cortex. Although some fold changes for individual DEmiRNA TSEAs are greater than one, the Benjamini–Hochberg correction showed no significance.

**Figure 10:**
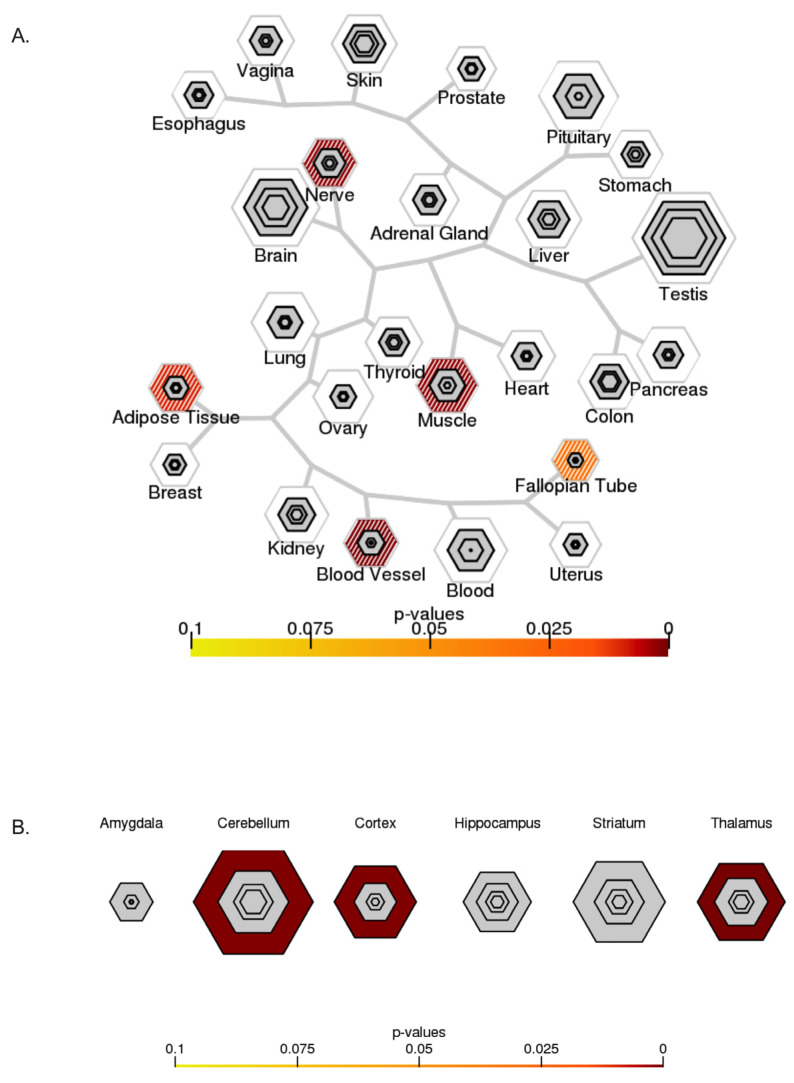
Specific expression analysis. (**A**) TSEA based on GTEx (http://genetics.wustl.edu/jdlab/tsea/#, accessed on 6 June 2023). Significant enrichment in nerve, muscle, blood vessels, and adipose tissues. (**B**) CSEA based on data from the Brainspan collection (http://genetics.wustl.edu/jdlab/csea-tool-2/, accessed on 6 June 2023). Significant enrichment in the cerebellum, cortex, and thalamus.

The TSEA for targets of DEmiRNAs based on RNA-Seq data from GTEx (Figure 10A) showed significant enrichment in the nerve, muscle, blood vessels, and adipose tissues. The *p*-value suggested a strong association in nerve, muscle, and blood vessel tissues.

CSEA for human brain cells and DEmiRNA targets (Figure 10B) showed significant enrichment in the cerebellum, cortex, and thalamus. Enrichment for CSEA was qualified as significant based on the *p*-value.

## 3. Discussion

MT is a recently developed treatment and has been used as a very efficient therapy for large vessel occlusion stroke. Currently, RT-PA is the main method of treating cerebral infarction. However, due to the short time window (4.5 h), its application is restricted to only a limited number of patients receiving thrombolytic therapy. Endovascular treatment (i.e., MT) increases the recanalization rate of occluded large vessels and prolongs the time window for stroke intervention (up to 24 h) compared to rt-PA. The biological mechanism behind its effectiveness has not yet been fully explored. Various miRNAs have been shown to be elevated or decreased in a stroke. These studies have been mostly advantageous for the prognosis or diagnosis of strokes, but none of them focused on miRNA relevance in stroke therapy effectiveness, its role in patient enrollment to a certain type of endovascular recanalization treatment, or the patient’s recovery rate. More comprehensive studies investigating the miRNA profile are needed to evaluate the effects of reperfusion treatment.

The research project presented here aimed to show whether the reperfusion therapy affected miRNA between the beginning and the end of the acute post-stroke phase, and we believe our preliminary results help to fill a significant knowledge gap. MiRNAs are a representative class of non-coding RNA molecules that mediate neurological alterations before, during, and after an ischemic stroke. Several miRNAs have been proposed as potential biomarkers for ischemic stroke to support the process of stroke risk assessment and the early detection of the disease [17,18]. However, we know very little about the impact of therapy on the miRNA profile and recovery prognosis. In this paper, we integrated preliminary miRNA NGS data from stroke patients after reperfusion treatment with detailed bioinformatics analyses. We identified 30 DEmiRNAs (*p* < 0.05), but after Bonferroni correction, we narrowed down the potential DEmiRNAs to the following 3: miR-9-3p, miR-9-5p, and miR-129-5p. The qRT-PCR validation confirmed the significant downregulation of identified miR-9-3p and miR-9-5p but did not obtain statistical significance for miR-129-5p. Human studies on serum from stroke patients [19], serum exosomes [20], or cerebral spinal fluid (CSF) [21] found elevated levels of miR-9-5p in acute ischemic stroke patients compared to those in healthy individuals. Wang et al. found a pronounced correlation between miR-9-5p upregulation and poor outcomes in patients after stroke. Increased miR-9 has been identified as a potential biomarker for diabetes complicated with stroke [22]. CSF shows elevated levels of miR-9-3p and miR-9-5p after subarachnoid hemorrhage, and these miRNAs are associated with a poor neurological outcome of delayed cerebral ischemia [23]. The analysis of serum from patients after traumatic brain injury (TBI) [24] revealed increased levels of miR-9-3p after TBI compared to the controls. Bioinformatic analysis revealed that miR-9-3p was significantly enriched in the brain compared to other tissues in patients with TBI [24]. In another study, elevated serum levels of exosomal miR-9 were positively correlated with the stroke severity scale and infarct volume [20]. Beske et al. showed that increased plasma levels of miR-9-3p were associated with unfavorable neurological outcomes following out-of-hospital cardiac arrest, and they reported peak levels of miR-9-3p 48 h after cardiac arrest [25]. The temporal analysis of altered miRNA in human stroke showed that miR-129-5p was upregulated and unique to the post-stroke recovery period [26]. We believe that the downregulated expression of miR-9 family members via reperfusion treatment confirms that miR-9-3p and miR-9-5p may have a functional value. Based on the functional analysis we performed here, we suggest that reperfusion treatment itself by reducing the levels of miR-9-3p and miR-9-5p affects the dependent metabolic pathways engaged in neuroprotection and, therefore, may influence the neuroprotective mechanisms in stroke patients.

Target prediction analysis revealed that DEmiRNAs regulate the following four common genes: *CREB5*, *OTUD7B*, *PCDH7*, and *PHIP*. *CREB5* is a cyclic AMP (cAMP)-responsive element binding protein (CREB) that belongs to the family of leucine zipper transcription factors. *CREB5* is specific for brain tissue (white matter) and is expressed in many regions of the brain, such as the thalamus, basal ganglia, hypothalamus, or medulla oblongata. Additionally, *CREB5* is found in various neuronal cells but mainly in oligodendrocytes. In the nervous system, growth factors (e.g., NGF, BDNF), hypoxia, oxidative, or glutamate stressors trigger the robust phosphorylation of CREB and CRE-mediated genes in neurons, which regulate a wide range of processes, including the proliferation, growth, and survival of neuronal precursors, and the synaptic connectivity of developing neurons [27,28]. The list of CREB target genes includes genes that control neurotransmission, cell morphology, signal transduction, transcription, and metabolism. Brain damage in stroke eliminates the somatosensory body map in the brain, and recovery from stroke involves the reorganization of the surviving cortical areas in adjacent motor and ectopic somatosensory regions [29,30,31]. Treadmill exercise improved short-term memory via the ERK-Akt-CREB-BDNF signaling pathway and resulted in the inhibition of apoptosis in the hippocampus of ischemia-affected gerbils [32]. CREB takes part in learning and memory consolidation through its involvement in adult hippocampal neurogenesis [33]. The downregulation of DEmiRNAs by reperfusion treatment may accelerate the CREB-mediated remapping mechanisms of sensorimotor functions associated with better recovery in human stroke [34]. Inflammation is another important factor in brain ischemia. Cerebral ischemic injury and the reperfusion of blood flow cause an inflammatory cascade, including oxidative stress, excitotoxicity, inflammatory cell infiltration, and the release of toxic inflammatory mediators that further contribute to neural tissue damage and cell death. CREB5 is associated with the immune system, where it plays various roles. CREB primarily promotes anti-inflammatory immune responses through the inhibition of NF-κB functions, induction of IL-10, and generation of Tregs. However, depending on the context, these responses can have a protective or pathogenic effect on the tissue [35]. We found that CREB5 is enriched in neutrophils, but its detailed function in these cells is still unknown. Though another member of the CREB family, CREB1 is responsible for neutrophil activation and pro-inflammatory cytokine production [36]. Neutrophils play a significant role in post-stroke pathology, where they promote blood–brain barrier disruption, cerebral edema, cellular injury, and neurological impairment. Anti-neutrophil therapy targets neutrophil activation, recruitment, and adhesion, as well as the release of proteases, ROS, and cytokines. However, early human studies face challenges, suggesting that the selective targeting of neutrophils may be required [37,38]. Several properties of neutrophils are protective, and thus, their antimicrobial, anti-inflammatory, and neuroprotective functions may be important to preserve tissue remodeling and repair during nerve cell recovery [39] in patients with stroke [40].

The next DEmiRNA target we discovered was the OUT domain-containing 7B (*OTUD7B*), called Cezanne, as a multifunctional deubiquitylate [41]. OTUD7B plays a diverse role in cancer and vascular diseases. Similarly, OTUD7B controls many important signaling pathways, including the inhibition of the NF-KB-mediated inflammatory response and restraining pro-inflammatory transcription in response to TNF receptor (TNFR) signaling [42,43]. In cardiovascular research, Cezanne has been implicated in scar formation, cell survival, the regulation of hypoxia, arterial remodeling, and neovascularization [44]. In the penumbra region, many neurons undergo reversible degeneration because of the supply of collateral circulation. This process provides the possibility to rescue the neurons and neurovascular unit via reperfusion treatment. The immediate restoration of local blood flow promotes the formation of new blood vessels in the ischemic region, which is not only necessary to rescue degenerated neurons but also provides a good microenvironment for neural stem cell survival, proliferation, and remodeling for functional repair. However, not much is known about the role of Cezanne in cerebral ischemic injury. Recently, Cheng et al. investigated the role of Cezanne-SIRT6-DNA DSB signaling pathways in I/R-induced ischemic brain injury in rats. The inhibition of Cezanne increased SIRT6 levels and conferred neuroprotection after cerebral ischemia injury in rats and in cultured neurons after OGD insult [45]. However, clinical research data showed that the prognosis of patients with a high density of new capillaries in the brain region affected by cerebral ischemia injury is significantly better than that of patients with a low density of new capillaries [46].

In the penumbra region, many neurons undergo reversible degeneration because of the supply of collateral circulation. This process provides the possibility to rescue the neurons and neurovascular unit by reperfusion treatment. The immediate restoration of local blood flow promotes the formation of new blood vessels in the ischemic region, which is not only necessary to rescue degenerated neurons but also provides a good microenvironment for neural stem cell survival, proliferation, and remodeling for functional repair. The prognosis of patients with a high density of new capillaries in the brain region affected by cerebral ischemia injury is significantly better than that of patients with a low density of new capillaries [46].

Another DEmiRNA target was *PCDH7*, which belongs to the non-clustered protocadherin PCDHδ1 subfamily and is termed brain–heart (BH)-protocadherin due to its predominant expression in the brain and heart [47]. In the brain, PCDH7 is produced in neurons and astrocytes [48], where it modulates axon/dendrite morphology. At the molecular level, PCDH7 is present in the excitatory synaptic cleft [49,50,51,52,53]. Although most studies on PCDH7 have focused on its role in cancer [50], some studies in recent years have linked PCDH7 to central nervous system disorders [53]. MeCP2 binds to the promoter region of *PCDH7* and downregulates its mRNA level, suggesting that the dysregulation of these molecules may be related to the neuronal and synaptic dysfunction observed in the brains of patients with Rett syndrome [54]. Genome-wide association studies have linked *PCDH7* to epilepsy, shorter sleep [55], and antipsychotic treatment responses in schizophrenic patients [56]. In hypertensive African Americans who suffer from a higher stroke burden due to hypertension, *PCDH7* is a plausible genetic determinant for stroke incidence [57].

The last target of DEmiRNA identified in this study was the *PHIP* gene, which encodes the pleckstrin homology domain interacting protein, which is involved in multiple biological processes, including cancer pathogenesis [58,59,60], cell cycle control [61] and metabolism [62]. Studies on *PHIP*-mutant mice [63] and mouse embryonic stem cells demonstrated that PHIP is dispensable for neurogenesis but is essential for postnatal growth and survival. However, *PHIP*’s chromatin binding is disrupted in neurodevelopmental disorders [64]. Loss-of-function mutations in the *PHIP* gene are associated with the neurodevelopmental disorder Chung–Jansen syndrome [65], which includes dysmorphic features, cognitive dysfunction, aberrant behavior, childhood-onset obesity, and severe childhood obesity related to the inhibition of pro-opiomelanocortin (POMC) expression: a neuropeptide that suppresses appetite [62].

Further tissue (TSEA) and cell (CSEA) enrichment analyses demonstrated that DEM targets are specific to brain cells in the cerebellum, cortex, and thalamus. The GO analysis of BPs and CCs revealed that targets were involved in neurogenesis (e.g., the generation of neurons) and neuronal differentiation (e.g., CC morphogenesis and neuron development). Analyses performed using the HALLMARK and PANTHER databases are consistent with stroke pathology. HALLMARK showed significant enrichment for DEmiRNAs in several pathways, including hypoxia [66], inflammation [67] (HALLMARK: Interleukin 2 STAT 5 signaling), the blood–brain barrier [68] (HALLMARK: apical surface, apical junction), cell growth and metabolism [69] (HALLMARK: PI3K AKT mTOR signaling), and cell proliferation [70] (HALLMARK: Mitotic spindle). PANTHER scores overlapped some of the above-mentioned metabolic pathways discovered in the GO and HALLMARK databases (e.g., axon guidance mediated by netrin). Several enrichment scores were similar for the PANTHER, GO, and HALLMARK databases, including the p38 MAPK pathway [71], RAS pathway [72], p53 pathway [73], PDGF signaling pathway [74], endothelin signaling pathway [75], integrin signaling pathway [76], TGF-beta signaling pathway, and angiogenesis [77].

KEGG enrichment analyses for DE genes revealed significant metabolic pathways related to longevity-regulating signaling pathways, which encompass genes regulating autophagy, mitochondrial activity, or oxidative stress and may trigger cellular senescence pathways, leading to irreversible cellular arrest. Conversely, DE genes retain the potential for self-renewal and differentiation by activating pathways that regulate stem cell pluripotency, focal adhesion, and the actin cytoskeleton in the nervous system. The prevailing conclusion is that stroke is a polygenic condition. Reperfusion treatment affected the miRNA profile of stroke patients and elicited the expression of targets commonly associated with dilated cardiomyopathy, cancer (hepatocellular carcinoma and gastric cancer), and human T-cell leukemia virus 1. These diseases are risk factors for stroke and indicators of poor post-stroke outcomes. Cardiomyopathy, associated with the macro- and microstructural remodeling of the heart cavities, affects systemic hemodynamic factors and could be the source of systemic embolism. Neoplasm disease is associated with a pro-thrombotic state and is one of the leading causes of embolic stroke. The association between brain ischemia and cancer is multifactorial and bidirectional. Furthermore, the mechanism of brain ischemia in cancer patients may be polyetiological. Tumors can release circulating microparticles into the bloodstream and increase the concentration of procoagulant factors, including factor X. Tumors can release mucins that activate platelets and endothelial cells through the binding of P- and L-selectin [78]. Cancers stimulate neutrophils to release de-condensed chromatin, forming neutrophil extracellular traps (NETs), which promote inflammation and thrombosis [79]. In particular, brain tumors can overexpress podoplanin, which is a transmembrane sialoglycoprotein and a potent activator of platelet aggregation [80].

### Limitations

Our study has a few limitations. First, the limited number of patients (total n = 43) and the small group size of the validated group (n = 38) could be responsible for the non-significant outcome for miR-129-5p or lack of correlation between the levels of miRNAs on day 10 and the patient’s functional outcome on day 90 in the mRS scale. The individual variations in patients, like the patient’s medication dosage, economic situation, rehabilitation, and the patient’s mental condition between day 10 and day 90 may significantly affect the functional outcome evaluation on day 90. As a result, it may mask the biological effect of tested miRNA on the patient’s functional outcome in the presented preliminary study. Therefore, further studies should be performed on a bigger cohort of patients, together with subgrouping patients according to their TICI (thrombolysis in cerebral infraction) scores. Second, the target estimation research was based on the recently published data package but did not include the most recent studies. The estimated targets for miRNA 9-3p and miR9-5p were enriched by the analysis of the OMIC databases and need further in situ revalidation. Therefore, further investigations are needed to demonstrate the importance of reperfusion treatment for molecular processes in ischemic cerebral tissue.

## 4. Materials and Methods

This study included patients hospitalized in the Upper Silesian Medical Center of the Silesian Medical University in Katowice between 2020 and 2022 due to stroke. The patients suffered from an ischemic stroke due to large vessel occlusion and were treated with reperfusion treatment within six hours of ischemic stroke onset.

### 4.1. Study Population 

During the study period, 443 stroke patients were treated with reperfusion therapy (MT and/or RT-PA) at our Comprehensive Stroke Center. Among these patients, we selected a group of 63 individuals. Based on the inclusion criteria, we initially included 31 patients in the study and finally qualified 5 patients for the NGS study (Table 1). Later, we qualified an additional 38 patients for the RT-qPCR study (Table 2).

Inclusion criteria are as follows: (1) 50–85 years of age, (2) patient’s first ever symptomatic ischemic stroke diagnosed according to the WHO definition and head CT and/or MRI result, (3) informed consent to participate in the study (with limitations in verbalizing their consent, a written declaration was provided by two people: a family representative and/or staff member uninvolved in the study’s course), (4) a pre-stroke status of 0–2 mRankin, (5) no history of intracranial hemorrhage and no other severe and/or disabling neurological disorders, and (6) the onset of symptoms up to 24 h prior to study enrollment. Exclusion criteria are as follows: (1) hemorrhagic transformation of stroke lesion, (2) pregnancy, (3) alcohol abuse/chronic use of a psychostimulant, (4) chronic infection/active neoplastic disease, (5) brain tumor, (6) history of a transient ischemic attack (TIA) or stroke, (7) renal/hepatic failure, or (8) surgery in the last three months. Table 1 summarizes the characteristics of the patients included in the study.

### 4.2. Sampling of Serum

Blood samples (5 mL) were collected twice from each patient by venipuncture into serum separator tubes (BD) on days 1 and 10 after the stroke’s onset. After incubation at room temperature for 30–45 min to allow clotting, the samples were then centrifuged at 1940× *g* for 10 min at room temperature. The supernatant was collected and pipetted into aliquots (500 µL). The samples were stored at –80 °C until further NGS and qRT-PCR analyses.

### 4.3. Library Preparation and Sequencing of miRNA/Small RNA-Seq from Serum

The Exiqon Genomics Services performed RNA extraction from the serum and small RNA sequencing (Hilden, Germany; *n* = 5). For 5 patients, 2 samples of 500 µL serum aliquot tubes (10 in total) were shipped to the Qiagen center (Hilden, Germany). The qPCR assay evaluated the quality of the tested samples. First, the expression levels of the samples were tested to see if the miRNA expressions were within the expected range for the miRNA content (hsa-miR-103a-3p, hsa-miR-191-5p, hsa-miR-451a, hsa-miR-23a-3p, and hsa-miR-30c-5p miRNAs are expressed in biofluids such as serum and plasma). The samples were then screened for the inhibition of enzymatic reactions (spike in control UniSp6) and potential hemolysis (miR23a-miR451a) [81]. The expression levels of the samples were within the expected range of miRNA content, and no inhibition or hemolysis was observed. The preparation of the small RNA library was then performed using the QIASeq miRNA library kit, including unique molecular identifiers (UMIs) for Illumina NGS systems (performed at the Qiagen center, Hilden, Germany). The single-end sequencing of 75 bp reads (50 bp in target and 25 bp for UMIs) was performed at a depth of 20 M, with one sample/lane in the Illumina Next-Seq 550.

### 4.4. Quantification of miRNAs and Differential Expression Analysis of miRNA

The raw fastq files acquired from small RNA-Seq were first manually inspected with FastQC (v. 0.11.3) to check the overall quality of the sequencing data. The fastq files were then uploaded to the Qiagen Geneglobe data analysis center (DAC), which is the web platform to analyze data from Qiagen’s QIASeq NGS library kits (https://geneglobe.qiagen.com/in/analyze/ (accessed on 15 September 2019). The primary quantification of read counts in the DAC was performed through the following three steps: (i) trimming the 3′-adapter and low-quality bases using cutadapt, (ii) identifying the insert sequences and UMIs (reads with <16 bp insert sequences or <10 bp UMI sequences were discarded), and (iii) aligning the processed reads to the human reference genome GRCh38 with a sequential alignment strategy using bowtie (with a perfect match to miRBase mature, miRBase hairpin, non-coding RNA, mRNA, and other RNAs, and ultimately a second mapping to miRBase mature, where up to 2 mismatches were tolerated). The annotation of miRNAs was performed with miRBase (v. 21). After primary quantification, we performed a differential expression analysis for miRNAs with DESeq2 (v. 1.22.2) in the R environment (v. 3.5.3). Data were visualized with R (v. 3.5.3). The mean expression levels were used for subsequent analysis. Preliminary statistical analysis was performed using the ‘Exact Test’ for two group comparisons [82]. Group 1 comprised samples acquired from patients on their 1st day (<24 h) after stroke, and group 2’s samples were from the same patients on the 10th day after reperfusion treatment. Sample description denoted the time of sample collection. An odd number signified that the sample was collected on day 1 after the stroke, and even-numbered samples were collected on day 10. We selected preliminarily significant miRNAs based on the ‘Exact Test’ (*p* < 0.05) and excluded the miRNAs that did not present a change in the mean expression level (the fold change for miRNAs was equal to 0) from further analysis. We conducted additional statistical tests on the remaining data with the Bonferroni test (*p*-adjusted < 0.01). Further analysis was performed on miRNAs that met the criteria of the Bonferroni correction (DEmiRNAs).

### 4.5. DE-miRNAs Validaton

Total miRNA was extracted from serum plasma samples of 38 stroke patients treated with reperfusion treatment using the GeneMATRIX Universal RNA/miRNA Purification Kit (E3599; EURx) according to the product’s instructions. The synthesis of cDNA was performed using the miRCURY LNA RT Kit (339340; Qiagen) with a total reaction volume of 10 µL, including 0.5 µL of UniSp6 and 10 ng/µL of miRNA. The expression level of DE-miRNAs was detected using RT PCR Mix SYBR (2008; A&A Biotechnology, Gdańsk, Poland), with cDNA as a template and the miRCURY LNA miRNA PCR assay. The mix reaction contained 6 µL of cDNA, 7 µL of SYBR Green, 1 µL of the primer (Table 3), and 1 µL of ddH_2_O. A 2-step cycling qPCR protocol (95 °C for 2 min followed by 60 cycles at 95 °C for 10 s and 56 °C for 60 s) was conducted using the CFX Opus 96 Real-Time PCR System (Bio-Rad, Hercules, CA, USA). The relative expression of each miRNA was normalized to UniSP6 by a relative quantitative method. The miRNA primer sequence is presented in Table 3.

miRNA levels were measured relative to UniSp6 levels, which served as an internal control. Relative miRNA levels were calculated using the comparative Ct method and expressed using the Livak method as fold changes relative to control samples [83]. Next, we used one sample t-test (µ = 1) to assess the statistical significance of 2^−ΔΔCt^ values. Differences at *p* < 0.05 were considered to be statistically significant.

Additionally, we performed the Spearman correlation between the level of miR-9-3p or miR-9-5p and the functional status on day 90 following stroke as per the modified Rankin Scale (mRS) [14].

### 4.6. Target Estimation Based on NGS Analysis and Enrichment Analysis

Based on the DEmiRNAs, we performed the target estimation [84]. First, we created a list of DEmiRNA targets using the molecular signatures database (MsigDB, category = “C3”, subcategory = “MIR: MIRDB”) (v. 7.5.1) [85,86]. To assess the number of individual and shared targets for DEmiRNAs, a Venn diagram was constructed [87].

Assuming that the DEmiRNA targets were significant, we performed an enrichment analysis (ShinyGO 0.80) for which we used HALLMARK [82,88], Gene Ontology (GO) [89,90], the Protein Analysis Through Evolutionary Relationships (PANTHER) classification system [88,89], and the Kyoto Encyclopedia of Genes and Genomes (KEGG) [91,92] databases. The *p*-value for the enrichment analysis was calculated by comparing the observed frequency of an annotation term with the frequency expected by chance [93]. The cut-off for significant pathways affected by targets of DEmiRNAs was set at *p* < 0.05 [82,88]. In the following evaluation, a fold change greater than or equal to 2 was assumed to be significant, according to Fold-Change-Specific Enrichment Analysis (FSEA) [94].

### 4.7. Tissue-Specific Expression Analysis (TSEA) and Cell-Type-Specific Expression Analysis (CSEA)

In addition to enrichment through the HALLMARK, KEGG, PANTHER, and GO databases, we performed the tissue-specific analysis of DEmiRNAs targets based on the Human Protein Atlas and Genotype-Tissue Expression (GTEx) projects. First, we used RNA-seq data from the Human Protein Atlas project for tissue enrichment [95]. Second, we used GTEx to perform tissue-specific expression analysis (TSEA), where the list of targets affected by DEmiRNAs [96,97] was checked for overlapping transcripts enriched in a particular tissue using Fisher’s Exact Test with a Benjamini–Hochberg correction.

To find cell populations likely to be affected by altered DEmiRNA levels in the adult human brain, we performed cell-type-specific expression analysis (CSEA). In this analysis, we used data from the Brainspan collection to indicate transcripts enriched in specific regions of the human brain [98].

The interpretation of TSEA and CSEA plots is given as follows (): the dendrogram skeleton depicts an approximation of the hierarchical clustering of tissues based on gene expression. The size of the outer hexagon is proportional to the number of transcripts enriched in a particular tissue at the least stringent threshold of pSI < 0.05. The size of the concentric hexagons is proportional to the number of transcripts enriched in a particular tissue at the more stringent threshold (0.001, 0.001, 0.0001 from the outermost to the innermost). A heatmap color scheme is added at the appropriate hexagon to depict the significance of Fisher’s Exact Test. Note that any significance in the outermost hexagon is hashed to reflect that the transcript lists are less specific at this threshold [99].

## 5. Conclusions

We discovered a significant decrease in hsa-miR-9-3p and hsa-miR-9-5p expression during the acute phase of stroke in patients treated with reperfusion treatment. Bioinformatic analysis showed that the negative regulation of hsa-miR-9-3p and hsa-miR-9-5p can promote neuroregeneration in treated stroke patients. Thus, we believe our data advances knowledge on the biological mechanism behind the efficacy of reperfusion treatment and points to the miRNA involved in reperfusion treatment efficacy as well as their target genes *CREB5*, *OTUD7B*, *PCDH7*, and *PHIP*, and proteins encoded by these target genes in the neuroprotective role of reperfusion treatment in stroke treatment. Our findings respond to the urgent need to further investigate the significance of miRNA in the development of a treatment strategy for acute stroke intervention.

## Figures and Tables

**Table 1:** Characteristics of the patients included in the NGS study.

Parameter	Patients, n = 5
Age, mean., med. [ref.]	66.6, 64 [50–82]
Occluded artery	
MCA right	3 (60%)
ICA left	2 (40%)
rtPA	4 (80%)
OCSP_TACI	5 (100%)
TOAST_A	3 (60%)
TOAST_C	2 (40%)
MT	
Stent retriever	4 (80%)
Aspiration	1 (20%)
Stroke onset–groin puncture, mean [ref.] min.	270 [210–360]
TICI	
1	0
1a	0
2b	2 (40%)
3	3 (60%)
AF	2 (40%)
AH	5 (100%)
DM	2(40%)
CAD	2 (40%)
PAD	1 (20%)
LD	1 (20%)
RBC_1	4.32 × 10^6^/μL [4.00–5.00]
WBC_1	10.72 × 10^3^/μL [4.00–10.00]
Lymphocyte_1	1.89 × 10^3^/μL [1.00–4.50]
Neutrophile_1	8.39 × 10^3^/μL [2.00–6.14]
Basophile_1	0.03 × 10^3^/μL [0.00–0.10]
Eosinophile_1	0.08 × 103/μL [0.05–0.50]
PLT_1	197 × 103/μL [135–350]
HCT_1	35.88% [36.00–47.00]
Hb_1	12.24 g/dL [12.00–16.00]
Creatinine	0.57 mg/dL [0.51–0.95]
eGFR	89 mL/min/1.73 m^2^ [>60]
CRP	13.0 mg/l [<5.0]
RBC_10	4.20 × 10^6^/μL [4.00–5.00]
WBC_10	8.83 × 106/μL [4.00–10.00]
PLT_10	320 × 103/μL [135–350]
HCT_10	36.20% [36.00–47.00]
Hb_10	12.35 g/dL [12.00–16.00]
Smoking	1 (20%)
NIHSS_1 med. [ref.]	13 [5–19]
NIHSS_2	13 [6–18]
NIHSS_10	8 [0–16]
mRS_dis	3 [0–4]
mRS_3m	3 [0–5]
**Parameter**	**Patients, n = 5**
Age, mean., med. [ref.]	66.6, 64 [50–82]
Occluded artery	
MCA right	3 (60%)
ICA left	2 (40%)
rtPA	4 (80%)
OCSP_TACI	5 (100%)
TOAST_A	3 (60%)
TOAST_C	2 (40%)
MT	
Stent retriever	4 (80%)
Aspiration	1 (20%)
Stroke onset–groin puncture, mean [ref.] min.	270 [210–360]
TICI	
1	0
1a	0
2b	2 (40%)
3	3 (60%)
AF	2 (40%)
AH	5 (100%)
DM	2(40%)
CAD	2 (40%)
PAD	1 (20%)
LD	1 (20%)
RBC_1	4.32 × 10^6^/μL [4.00–5.00]
WBC_1	10.72 × 10^3^/μL [4.00–10.00]
Lymphocyte_1	1.89 × 10^3^/μL [1.00–4.50]
Neutrophile_1	8.39 × 10^3^/μL [2.00–6.14]
Basophile_1	0.03 × 10^3^/μL [0.00–0.10]
Eosinophile_1	0.08 × 10^3^/μL [0.05–0.50]
PLT_1	197 × 10^3^/μL [135–350]
HCT_1	35.88% [36.00–47.00]
Hb_1	12.24 g/dL [12.00–16.00]
Creatinine	0.57 mg/dL [0.51–0.95]
eGFR	89 mL/min/1.73 m^2^ [>60]
CRP	13.0 mg/l [<5.0]
RBC_10	4.20 × 10^6^/μL [4.00–5.00]
WBC_10	8.83 × 10^6^/μL [4.00–10.00]
PLT_10	320 × 10^3^/μL [135–350]
HCT_10	36.20% [36.00–47.00]
Hb_10	12.35 g/dL [12.00–16.00]
Smoking	1 (20%)
NIHSS_1 med. [ref.]	13 [5–19]
NIHSS_2	13 [6–18]
NIHSS_10	8 [0–16]
mRS_dis	3 [0–4]
mRS_3m	3 [0–5]

ICA—internal carotid artery, MCA—middle cerebral artery, rtPA—recombinant tissue plasminogen activator, OCSP—Oxfordshire Community Stroke Project, TACI—total anterior cerebral artery, TOAST—trial of ORG 10172 in acute stroke treatment, TOAST_A—atherosclerosis, TOAST_C—cor, MT—mechanical thrombectomy, AF—atrial fibrillation, AH—arterial hypertension, DM—diabetes mellitus, CAD—coronary artery disease, PAD—peripheral artery disease, LD—lipid disorders, RBC_1—red blood cells on the 1st day, RBC_10—red blood cells on the 10th day, WBC_1—white blood cells on the 1st day, WBC_10—white blood cells on the 10th day, PLT_1—platelets on the 1st day, PLT_10—platelets on the 10th day, HCT_1—hematocrit on the 1st day, HCT_10—hematocrit on the 10th day, Hb_1—hemoglobin on the 1st day, Hb_10—hemoglobin on the 10th day, eGFR_1—estimated glomerular filtration rate on the 1st day, eGFR_10—estimated glomerular filtration rate on the 10th day, CRP_1—C-reactive protein on the 1st day, CRP_10—C-reactive protein on the 10th day, NIHSS—National Institutes of Health Stroke Scale, NIHSS_1—NIHSS on the 1st day, NIHSS_2—NIHSS on the 2nd day, NIHSS_10—NIHSS on the 10th day, mRS—modified Rankin Scale, mRS_dis—mRS at discharge, and mRS_3m—mRS on the 90th day. For laboratory tests, values in the square [ ] brackets indicate reference values for the presented parameter. In other cases, the square [ ] brackets enclose the lowest and highest value for a given parameter assessed in patients included in the study.

**Table 2:** Characteristics of the patients included in the RT-qPCR study.

Parameter	Patients, n = 38
Age, mean., med. [ref.]	66.6, 64 [50–82]
Occluded artery	
MCA right	7 (18.42%)
MCA left	20 (52.63%)
ICA left	4 (10.53%)
PCA left	1 (2.63%)
VA right	1(2.63%)
No occlusion	5 (13.16%)
rtPA	28 (73.7%)
OCSP_TACI	12 (31.58%)
OCSP_PACI	21 (55.26%)
OCSP_LACI	3 (7.89%)
OCSP_POCI	2 (5.26%)
TOAST_A	14 (36.84%)
TOAST_C	11 (28.95%)
TOAST_S	11 (28.95%)
TOAST_U	2 (5.26%)
MT	
Stent retriever	18 (47.37%)
Aspiration	8 (21.05%)
Stroke onset–groin puncture, mean [ref.] min.	282 [145–360]
TICI	
0	2 (5.26%)
2C	1 (2.63%)
3	23 (60.53%)
AF	16 (42.11%)
AH	36 (94.74%)
DM	16 (42.11%)
CAD	17 (44.74%)
PAD	14 (36.84%)
LD	23 (60.52%)
RBC_1	4,03 × 10^6^/μL [4.00–5.00]
WBC_1	8,44 × 10^3^/μL [4.00–10.00]
Lymphocyte_1	1.36 × 10^3^/μL [1.00–4.50]
Neutrophile_1	6.09 × 10^3^/μL [2.00–6.14]
Basophile_1	0.03 × 10^3^/μL [0.00–0.10]
Eosinophile_1	0.04 × 10^3^/μL [0.05–0.50]
PLT_1	201 × 103/μL [135–350]
HCT_1	36.95% [36.00–47.00]
Hb_1	12.9 g/dL [12.00–16.00]
creatinine	0.87 mg/dL [0.51–0.95]
eGFR	83 mL/min/1.73 m2 [>60]
CRP	9.4 mg/L [<5.0]
RBC_10	4.06 × 106/μL [4.00–5.00]
WBC_10	7.8 × 106/μL [4.00–10.00]
PLT_10	228 × 103/μL [135–350]
HCT_10	36.75% [36.00–47.00]
Hb_10	12.6 g/dL [12.00–16.00]
Smoking	12 (31.59%)
Statins	15 (39.5%)
ASA	9 (23.7%)
DAPT	3 (7.9%)
VKA	5 (13.16%)
NIHSS_1 med. [ref.]	12 [1–28]
NIHSS_2	4.5 [0–28]
NIHSS_10	2 [0–24]
mRS_dis	2 [0–6]
mRS_3m	2 [0–6]

ICA—internal carotid artery, MCA—middle cerebral artery, PCA—posterior cerebral artery, rtPA—recombinant tissue plasminogen activator, OCSP—Oxfordshire Community Stroke Project, TACI—total anterior cerebral artery, PACI—partial anterior cerebral artery, LACI—lacunar infarct, POCI—posterior circulation infarct, TOAST—trial of ORG 10172 in acute stroke treatment, TOAST_A—atherosclerosis, TOAST_C—cardioembolism, TOAST_S—small vessel occlusion, TOAST_U—unknow/others origin of stroke, MT—mechanical thrombectomy, AF—atrial fibrillation, AH—arterial hypertension, DM—diabetes mellitus, CAD—coronary artery disease, PAD—peripheral artery disease, LD—lipid disorders, RBC_1—red blood cells on the 1st day, RBC_10—red blood cells on the 10th day, WBC_1—white blood cells on the 1st day, WBC_10—white blood cells on the 10th day, PLT_1—platelets on the 1st day, PLT_10—platelets on the 10th day, HCT_1—hematocrit on the 1st day, HCT_10—hematocrit on the 10th day, Hb_1—hemoglobin on the 1st day, Hb_10—hemoglobin on the 10th day, eGFR_1—estimated glomerular filtration rate on the 1st day, eGFR_10—estimated glomerular filtration rate on the 10th day, CRP_1—C-reactive protein on the 1st day, CRP_10—C-reactive protein on the 10th day, Statins prior to stroke event (atorvastatin or rosuvastatin), ASA—aspirin, DAPT dual antiplatelet therapy (clopidogrel and aspirin), VKA—warfin, NIHSS—National Institutes of Health Stroke Scale, NIHSS_1—NIHSS on the 1st day, NIHSS_2—NIHSS on the 2nd day, NIHSS_10—NIHSS on the 10th day, mRS—modified Rankin Scale, mRS_dis—mRS at discharge, and mRS_3m—mRS on the 90th day. For laboratory tests, values in the square [ ] brackets indicate reference values for the presented parameter. In other cases, the square [ ] brackets enclose the lowest and highest value for a given parameter assessed in patients included in the study.

**Table 3:** miR-9-3p, miR-9-5p, miR-129-5p primer sequences.

miRNA	Sequence
miR-9-3p	5′AUAAAGCUAGAUAACCGAAAGU
miR-9-5p	5′UCUUUGGUUAUCUAGCUGUAUGA
miR-129-5p	5′CUUUUUGCGGUCUGGGCUUGC

## Data Availability

Data is contained within the article.
